# When Genomic Scars Outlive Genomic Vulnerability: Dynamic DNA Damage Repair in Cancer Evolution and Therapeutic Resistance

**DOI:** 10.3390/genes17101250

**Published:** 2026-10-10

**Authors:** Alessandro Ottaiano, Francesco Sabbatino, Anna Chiara Carratù, Marco Cascella, Antonio Barone, Francesco Perri, Andrea Belli, Alfredo Columbro, Francesco Izzo, Mariafelicia Valeriani, Marco Correra, Guglielmo Nasti, Giovanni Savarese

**Affiliations:** 1 Istituto Nazionale Tumori di Napoli, IRCCS “G. Pascale”, Via Mariano Semmola, 80131 Naples, Italy; annachiara.carratu@istitutotumori.na.it (A.C.C.); f.perri@istitutotumori.na.it (F.P.); a.belli@istitutotumori.na.it (A.B.); f.izzo@istitutotumori.na.it (F.I.); m.correra@istitutotumori.na.it (M.C.); g.nasti@istitutotumori.na.it (G.N.); 2 Department of Medicine, Surgery and Dentistry, University of Salerno, Via Salvador Allende, 84081 Baronissi, Italy; fsabbatino@unisa.it (F.S.); mcascella@unisa.it (M.C.); mvaleriani@studenti.unisa.it (M.V.); 3 AMES Centro Polidiagnostico Strumentale, Via Padre Carmine Fico 24, 80013 Casalnuovo di Napoli, Italy; antonio.barone@centroames.it (A.B.); alfredo.columbro@centroames.it (A.C.); giovanni.savarese@centroames.it (G.S.)

**Keywords:** DNA damage repair, homologous recombination deficiency, genomic scars, *BRCA1/2*, PARP inhibitors, circulating tumor DNA, therapeutic resistance, precision oncology

## Abstract

Genomic biomarkers are increasingly used to identify therapeutically exploitable defects in DNA damage repair (DDR). However, genomic alterations and mutational scars largely represent the evolutionary history of a tumor, whereas therapeutic sensitivity depends on its current functional state. This distinction becomes particularly relevant during cancer progression and treatment, when selective pressure may reshape DNA repair capacity without erasing the genomic footprints generated by previous repair deficiency. Here, we examine the emerging concept of genomic scar–functional state discordance, whereby persistent genomic evidence of defective DNA repair may coexist with partial or complete restoration of repair competence. Multiple mechanisms may contribute to this transition, including secondary or reversion mutations, restoration of homologous recombination, replication fork stabilization, compensatory pathway activation, clonal selection, and epigenetic reprogramming. Conversely, initially repair-proficient tumors may acquire new DDR vulnerabilities during evolution, emphasizing the bidirectional and dynamic nature of genome maintenance. These processes challenge the assumption that a single genomic assessment can reliably define a stable therapeutic vulnerability throughout the disease course. We discuss how longitudinal approaches integrating tumor genomics, circulating tumor DNA, mutational signatures, epigenomic information, and functional measures of DNA repair could distinguish historical genomic damage from active biological dependency. Particular attention is given to gastrointestinal malignancies, including pancreatic, colorectal, gastric, and biliary tract cancers, in which DDR alterations are increasingly investigated but their predictive interpretation remains heterogeneous. We propose that precision oncology should progressively move from the concept of a static genomic biomarker toward that of a dynamic genomic state, in which therapeutic vulnerabilities are periodically reassessed as tumors evolve under treatment pressure.

## 1. Introduction

Maintenance of genomic integrity relies on a complex and interconnected network of DNA damage response and repair (DDR) pathways that continuously detect DNA lesions, coordinate cell cycle checkpoints, and restore damaged DNA. Disruption of these mechanisms is a major driver of genomic instability and cancer evolution, but may simultaneously create therapeutically exploitable dependencies. Homologous recombination deficiency (HRD) represents the most established example of this biological duality: functional impairment of BRCA1, BRCA2, PALB2, or other proteins involved in homologous recombination repair (HRR) promotes the accumulation of genomic damage while potentially increasing sensitivity to platinum compounds and poly(ADP-ribose) polymerase (PARP) inhibitors [1,2]. Consequently, identification of HRD has become an important component of precision oncology, although its optimal biological definition and assessment remain incompletely resolved [1,2,3]. HRD can be investigated at different biological levels. Beyond pathogenic alterations in individual HRR genes, prolonged defective DNA repair generates cumulative genomic footprints, including loss of heterozygosity, telomeric allelic imbalance, large-scale state transitions, characteristic mutational signatures, and structural rearrangement patterns [1,3]. These features constitute the basis of genomic scar assays and genome-wide classifiers developed to infer previous or ongoing HRD from the architecture of the tumor genome [1,2,3]. However, the very persistence that makes genomic scars detectable also creates an important biological limitation. Genomic scars primarily record the consequences of past DNA repair failure, whereas therapeutic sensitivity depends on the repair capacity that cancer cells possess when treatment is administered. Accordingly, genomic evidence of HRD and current functional HR competence should not necessarily be considered interchangeable biological states [3,4]. Functional approaches based on *RAD51* foci increasingly support this distinction, identifying discrepancies between genomic HRD classification and the actual capacity of tumor cells to perform HR [4,5].

This distinction becomes particularly relevant during cancer evolution. Therapeutic pressure can select subclones capable of restoring DNA repair through secondary or reversion mutations in *BRCA1/2*, alterations in regulators of DNA-end resection, restoration of *RAD51* loading, replication fork protection, and other compensatory mechanisms [6,7,8]. In classical genetic terminology, these compensatory events can be broadly distinguished as intragenic suppressors, when functional restoration arises through secondary changes within the originally altered gene, and extragenic suppressors, when alterations in other genes compensate for the primary defect. Recent longitudinal analyses provide compelling evidence that this transition can occur without erasing the genomic consequences of the preceding repair-deficient state. In *BRCA1*-, *BRCA2*-, or *PALB2*-mutated metastatic breast cancers progressing after HRD-targeted therapy, *BRCA1/2* reversions were detected in 60% of patients; importantly, tumors carrying reversions retained HRD-associated mutational signatures while demonstrating functional homologous recombination by *RAD51* assessment [6]. Similarly, extensive longitudinal analysis of tissue and circulating cell-free DNA has demonstrated convergent emergence of multiple independent *BRCA2* reversions under therapeutic pressure, illustrating how strongly treatment can select for restoration of repair competence [7].

These observations challenge a central assumption of biomarker-driven oncology: that a molecular vulnerability identified at one time point remains biologically stable throughout the disease course. Conversely, tumors initially proficient in DNA repair may acquire new DDR defects during progression, making the relationship between genomic alteration, genomic scar, functional repair capacity, and therapeutic response intrinsically dynamic. This problem is increasingly relevant beyond breast and ovarian cancers. In gastrointestinal malignancies, recent studies have identified potentially actionable HRD phenotypes in pancreatic, colorectal, gastric, and biliary tract cancers, while simultaneously demonstrating substantial heterogeneity between HRR gene alterations, genomic HRD, and sensitivity to platinum or PARP inhibition [9,10,11,12].

In this narrative review, we examine the emerging concept of genomic scar–functional state discordance, whereby the genomic record of previous DDR deficiency may persist after partial or complete restoration of repair competence. We also consider the opposite evolutionary trajectory, in which new DDR vulnerabilities emerge during tumor progression. Finally, we discuss how longitudinal integration of tumor genomics, circulating tumor DNA, mutational signatures, epigenomic information, and functional repair assays could help distinguish the historical record of genomic damage from the current biological state of DNA repair, shifting DDR-based precision oncology from the interpretation of a static genomic biomarker toward the assessment of a dynamic genomic and functional state.

## 2. Genomic Scars and Functional DNA Repair: Two Different Dimensions of Tumor Biology

**Figure 1:**
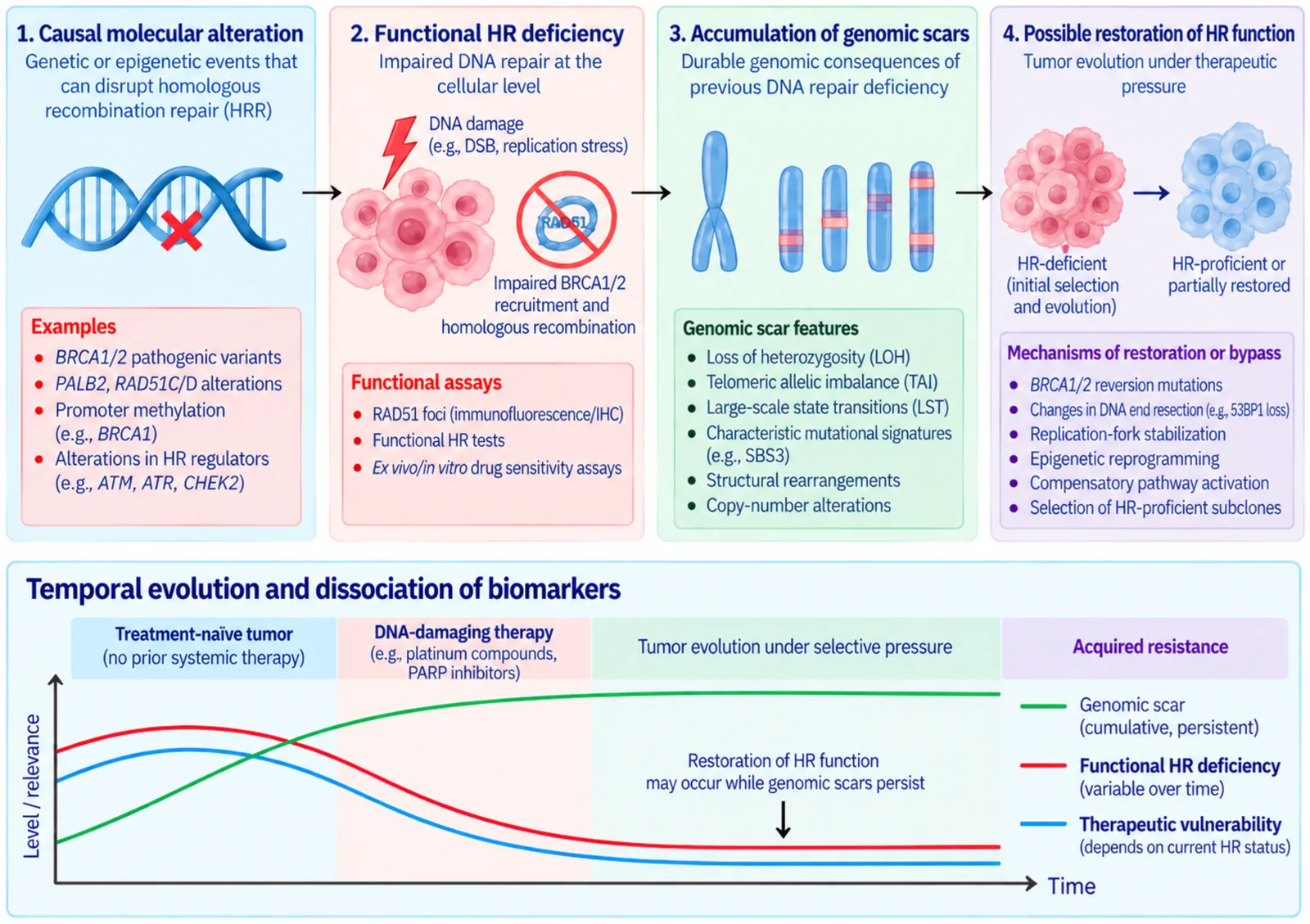
Genomic scars and functional DNA repair as distinct temporal dimensions of HRD. Causal genetic or epigenetic alterations may induce functional homologous recombination (HR) deficiency and the progressive accumulation of persistent genomic scars. Under therapeutic selective pressure, HR function may subsequently be restored or bypassed, reducing therapeutic vulnerability while genomic scars remain detectable. Figure created with the assistance of ChatGPT 5.5 (OpenAI). The scientific content, final review, and validation were entirely performed by the Authors.

The concept of HRD illustrates a fundamental distinction between the historical consequences of defective DNA repair and the current functional capacity of a cancer cell to repair DNA. HRD is not a single molecular event but rather a biological phenotype that may originate from heterogeneous genetic and epigenetic alterations affecting *BRCA1*, *BRCA2*, *PALB2*, *RAD51* paralogs, and other components or regulators of HRR [13,14]. When HRR is impaired, DNA double-strand breaks and replication-associated lesions cannot be accurately resolved through HR, forcing cells to rely increasingly on alternative and frequently error-prone repair mechanisms. Repeated cycles of defective repair progressively reshape the cancer genome, producing characteristic patterns of allelic imbalance, loss of heterozygosity (LOH), telomeric allelic imbalance (TAI), large-scale state transitions (LST), single-nucleotide substitutions, small insertions and deletions, and structural rearrangements [13,14,15]. These cumulative abnormalities constitute what are commonly referred to as genomic scars. LOH reflects genomic regions in which one parental allele has been lost; TAI identifies regions of allelic imbalance extending to a telomere without crossing the centromere; and LSTs quantify large chromosomal transitions between adjacent genomic segments, reflecting large-scale structural instability. These measures can be derived from genome-wide copy-number and allelic profiles and may be integrated into a composite genomic instability or HRD score [14]. Beyond these classical chromosomal scars, HRD can leave additional genomic footprints, including characteristic single-base substitution, insertion/deletion, rearrangement, and copy-number signatures, which can be captured by sequencing-based approaches [16]. Importantly, genomic scars are not equivalent to the molecular defect that originally generated them. Rather, they are the durable genomic consequences of having experienced a particular state of DNA repair deficiency over time. These biologically distinct but temporally interconnected dimensions of HRD—from the causal molecular alteration to functional repair deficiency and the subsequent accumulation of genomic scars—are schematically illustrated in Figure 1.

This distinction has important biological and clinical implications. Contemporary HRD assays interrogate different layers of this phenomenon. Some approaches identify potentially causative events, such as pathogenic *BRCA1/2* or other HRR gene alterations, whereas genomic scar assays quantify their downstream genomic consequences. Genome-wide mutational signature approaches extend this principle further by reconstructing characteristic patterns of mutagenesis attributable to defective HR [13,17]. These strategies have considerably expanded the ability to recognize HRD beyond germline or somatic *BRCA1/2* mutations and have demonstrated that an HRD-like genomic architecture may exist in tumors without an immediately identifiable canonical *BRCA* alteration [13,14,15,16,17]. However, all these approaches share an intrinsic temporal limitation: the genomic architecture observed at a given time is cumulative. A chromosomal rearrangement, segmental LOH event, or mutational signature generated during an earlier HR-deficient phase remains incorporated into the tumor genome even if the biological process responsible for generating it subsequently changes. In this sense, genomic scars function as an evolutionary archive rather than as a direct real-time measurement of DNA repair activity.

Functional assays interrogate a fundamentally different biological dimension. Among these, assessment of nuclear *RAD51* foci after DNA damage has emerged as one of the most promising strategies for determining whether tumor cells are currently capable of executing HRR [18,19,20]. *RAD51* recruitment to damaged DNA is a critical downstream step of HR, and its detection therefore provides information closer to the actual functional status of the pathway than the presence of an upstream genomic alteration or its historical genomic consequences. Recent studies have demonstrated clinically meaningful discordance between genomic HRD classifications and *RAD51*-based functional measurements, indicating that tumors carrying genomic evidence compatible with HRD may nevertheless retain—or recover—functional HR capacity [18,19,20]. Conversely, functional HR deficiency may occasionally be detected in tumors not classified as HRD by conventional genomic criteria. These observations indicate that genotype, genomic phenotype, and functional repair state represent related but non-identical biological variables.

The temporal dimension becomes particularly important under therapeutic selection. Platinum compounds and PARP inhibitors preferentially eliminate cells that remain dependent on defective HRR, thereby creating strong selective pressure for pre-existing or newly generated subclones capable of restoring sufficient DNA repair capacity [21,22,23]. A striking demonstration comes from longitudinal profiling of *BRCA1*-, *BRCA2*-, and *PALB2*-altered breast cancers progressing after HRD-directed therapies. Tumors acquiring *BRCA1/2* reversion events could retain mutational features associated with HRD while simultaneously showing evidence of restored HR by *RAD51*-based functional assessment [21]. Such observations provide direct support for a central premise of this review: a tumor can remain genomically scarred by HRD without remaining functionally HR-deficient. The apparent discrepancy disappears once the tumor is considered as an evolving biological system.

The same principle extends beyond *BRCA* reversions. Restoration of HRR may occur through changes in DNA-end resection; loss or alteration of proteins such as 53BP1, RIF1, or components of the Shieldin pathway; restoration of *RAD51* recruitment; epigenetic reprogramming; or selection of HR-proficient cellular populations [21,22,23,24]. Moreover, resistance to DNA-damaging therapy does not invariably require complete restoration of HR. Stabilization and protection of stalled replication forks can reduce the lethal consequences of replication stress in *BRCA*-deficient cells and thereby confer resistance while preserving at least part of the underlying HR-deficient genotype [25,26]. Thus, even the binary terminology of “HRD-positive” and “HRD-negative” may inadequately describe a biological continuum in which different components of genome maintenance can be independently lost, restored, bypassed, or compensated.

This temporal mismatch has a direct consequence for biomarker interpretation. A baseline tumor sample may accurately document a therapeutically relevant vulnerability at diagnosis yet become progressively less representative after multiple treatment lines and successive evolutionary bottlenecks. Conversely, the persistence of an HRD-associated genomic scar in a later specimen cannot, by itself, establish that the original vulnerability remains therapeutically exploitable. This distinction may be particularly important when genomic scar scores or mutational signatures are interpreted as predictors of sensitivity to PARP inhibition or platinum-based therapy. Their predictive value necessarily depends not only on whether HRD occurred during tumor evolution, but also on whether functionally relevant HR deficiency persists at the time therapeutic pressure is applied [13,18,21]. Accordingly, the clinically relevant question may need to evolve from “Does this tumor bear evidence of HRD?” toward “Is this tumor HR-deficient now?”

We therefore propose that DDR biomarkers should be interpreted across at least three interconnected but temporally distinct levels: the causal molecular alteration, representing the genetic or epigenetic event capable of disrupting repair; the genomic scar, representing the cumulative historical consequences of that disruption; and the current functional state, representing the repair capacity actually operating in the evolving tumor population. These layers may coincide in treatment-naïve disease, but therapeutic selection and clonal evolution can progressively uncouple them. Recognizing this separation provides the conceptual foundation for genomic scar–functional state discordance and, more broadly, for viewing DDR not as a fixed tumor attribute but as a dynamic phenotype whose therapeutic relevance can change throughout cancer evolution.

## 3. How Genomic Vulnerability Is Lost: Mechanisms of Functional DDR Restoration and Therapeutic Escape

**Figure 2:**
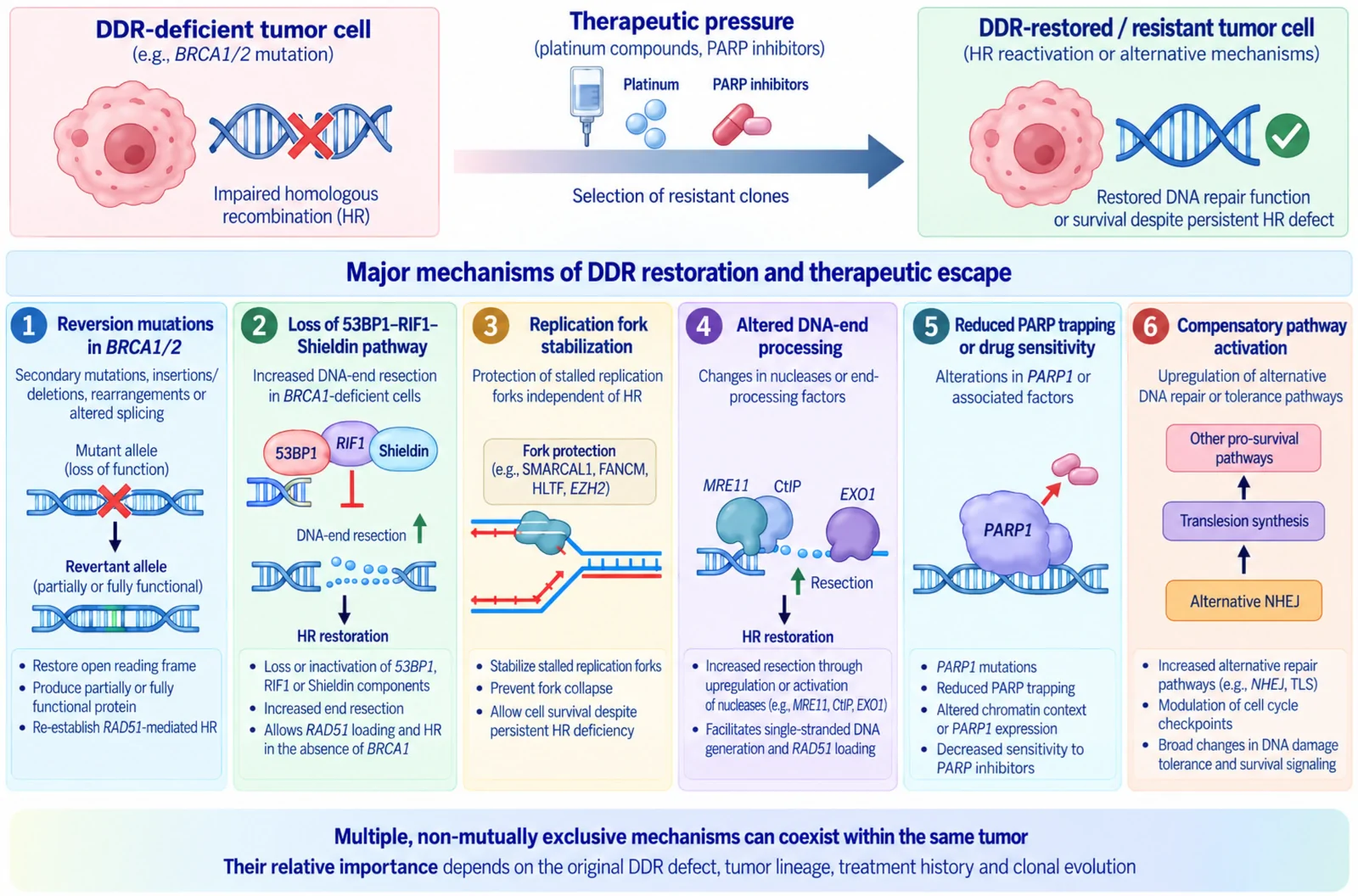
Mechanisms of functional DDR restoration and therapeutic escape. Therapeutic pressure from platinum compounds and PARP inhibitors selects resistant tumor clones through multiple, non-mutually exclusive mechanisms, including *BRCA1/2* reversions, restoration of DNA-end resection, replication fork stabilization, altered DNA-end processing, reduced PARP trapping, and compensatory pathway activation. These adaptations may restore homologous recombination or enable survival despite persistent HR deficiency. Figure created with the assistance of ChatGPT 5.5 (OpenAI). The scientific content, final review, and validation were entirely performed by the Authors.

The loss of a DDR vulnerability during cancer evolution does not require reversal of the genomic instability accumulated during tumor development. Rather, therapeutic resistance can emerge whenever cancer cells recover, bypass, or sufficiently compensate for the specific repair function on which treatment efficacy depends. This distinction is particularly evident in tumors exposed to platinum compounds and poly(ADP-ribose) polymerase inhibitors (PARPi), where prolonged therapeutic pressure creates a stringent evolutionary bottleneck favoring cells capable of tolerating DNA damage that was previously lethal. Several molecular routes can produce this phenotype, and their relative importance depends on the original DDR defect, tumor lineage, treatment history, and intratumoral clonal architecture. Importantly, these mechanisms are neither mutually exclusive nor biologically equivalent: some directly restore HR, whereas others preserve cell survival despite persistent HR deficiency by stabilizing replication intermediates, altering DNA-end processing, reducing PARP trapping, or modifying the balance between competing repair pathways [27,28]. The major mechanisms through which therapeutic pressure can promote functional DDR restoration, compensation, or therapeutic escape are summarized in Figure 2.

Among these mechanisms, secondary or reversion mutations in *BRCA1* and *BRCA2* provide the clearest genetic example of restoration of a previously lost repair function. Reversions can arise through secondary substitutions, insertions, deletions, intragenic rearrangements, or altered splicing that correct or circumvent the original pathogenic alteration and restore an open reading frame capable of producing a partially or substantially functional protein [29,30,31]. Their functional consequence is more important than restoration of the wild-type sequence itself: many revertant proteins remain structurally abnormal but retain sufficient activity to support *RAD51*-mediated HR and thereby reduce sensitivity to platinum and PARPi. Recent clinical studies indicate that this phenomenon is not exceptional under strong therapeutic selection. Longitudinal profiling of PARPi-resistant advanced breast cancers identified *BRCA1/2* reversions in a substantial proportion of resistant tumors and revealed previously underappreciated mechanisms, including intragenic deletions with intronic breakpoints and secondary mutations generating novel splice acceptor sites [28]. In advanced prostate cancer, analysis of samples collected during PARPi therapy similarly demonstrated reversions in the majority of *BRCA2*/*PALB2*-mutated tumors at the end of treatment, while tumors harboring homozygous *BRCA2* deletion could escape through selection of rare populations lacking the original homozygous deletion rather than through conventional sequence reversion [32]. These observations broaden the concept of “reversion”: evolutionary restoration of repair competence can occur either by molecular reconstruction of the damaged gene or by selection of a pre-existing cellular population in which the therapeutically relevant defect was never completely fixed.

The diversity of reversion events within individual patients further illustrates the strength of this selective pressure. Extensive multiregional tissue and circulating cell-free DNA analyses in a patient with *BRCA2*-mutated metastatic prostate cancer exposed to PARP inhibition identified ten distinct *BRCA2* reversions across ten metastatic sites, with most lesions harboring at least one independently acquired rescue event [30]. Rather than representing stochastic molecular noise, such convergent evolution indicates that restoration of *BRCA* function can confer sufficiently strong fitness advantage for different tumor populations to reach the same biological solution through independent genetic routes. The emergence of multiple reversions also challenges the representation of resistance by a single post-progression biopsy: different metastatic sites may restore repair through distinct molecular events, and their relative contribution to circulating tumor DNA (ctDNA) may vary considerably [30]. Consistently, reversions have been detectable in ctDNA before radiological progression in longitudinal studies, suggesting that molecular restoration of DDR can precede its clinically visible consequence [29]. Thus, resistance should be considered an evolving population-level process rather than a discrete molecular event occurring synchronously throughout the tumor burden.

*BRCA* sequence restoration, however, represents only one route toward therapeutic escape. In *BRCA1*-deficient cells, HR can be partially restored without restoring *BRCA1* itself through remodeling of DNA double-strand break end processing. The 53BP1–RIF1–Shieldin axis normally restricts DNA-end resection and favors non-homologous end-joining; loss of components of this pathway can increase resection, facilitate generation of single-stranded DNA, and permit downstream HR machinery to operate despite deficient *BRCA1* [27,33]. Loss-of-function alterations involving *TP53BP1*, *RIF1* and *PAXIP1* have now also been detected in clinical specimens from patients with acquired PARPi resistance, sometimes coexisting with *BRCA* reversions [29]. This coexistence is conceptually important because it argues against a simple one-tumor/one-resistance-mechanism model. Under continued selection, cancer populations may accumulate multiple solutions to the same therapeutic constraint, producing different degrees of repair restoration across spatially separated subclones. Moreover, these mechanisms are context-dependent: restoration of DNA-end resection can compensate for certain *BRCA1* defects but does not equivalently rescue loss of *BRCA2*, which acts downstream in *RAD51* loading and filament formation [27,33]. The precise position of the original lesion within the HR pathway therefore constrains the evolutionary routes available for functional rescue.

Therapeutic escape may also occur without canonical restoration of HR. Protection of stalled replication forks represents a particularly important alternative. *BRCA1/2*-deficient cells exhibit defective protection of stalled forks, allowing nucleolytic degradation of nascent DNA and contributing to the cytotoxicity of replication stress. Alterations that prevent recruitment or activity of nucleases at stalled forks can stabilize nascent DNA and increase survival despite persistence of the upstream HR defect [28,34]. Loss or modulation of proteins such as *PTIP*, *CHD4* and *EZH2* has been implicated in restoration of fork protection and resistance to PARPi or platinum agents [28,34]. This distinction has become increasingly relevant as mechanistic studies separate the different functions of BRCA proteins. Recent experimental analysis of patient-derived *BRCA2* reversion alleles, for example, showed that some revertant proteins restored homology-directed repair sufficiently to confer PARPi resistance despite remaining defective in replication fork protection, suggesting that restoration of individual *BRCA* functions can be uncoupled [35]. Emerging work on single-stranded DNA gaps similarly suggests that PARPi sensitivity cannot be reduced to a binary measurement of double-strand-break HR alone; suppression, protection, or filling of replication-associated gaps may represent an additional determinant of treatment response [28]. DDR restoration should therefore not be conceptualized as the simple reactivation of a single pathway, but as recovery of a minimum functional threshold of genome maintenance sufficient for survival under a specific therapeutic pressure.

Epigenetic plasticity adds another layer to this process. Some tumors acquire HRD through transcriptional silencing rather than irreversible gene disruption, most notably through *BRCA1* promoter methylation. Such states are potentially reversible: loss of promoter methylation or selection of cells with lower methylation can restore *BRCA1* expression and diminish sensitivity to PARPi or platinum therapy [27,36]. Alternative splicing and stabilization of hypomorphic BRCA proteins may similarly preserve sufficient residual function to support therapeutic resistance without returning the gene to its original configuration [27,36]. These mechanisms emphasize that DDR phenotype cannot always be inferred from DNA sequence alone. A tumor may retain the same nominal genomic alteration while changing transcription, protein abundance, pathway connectivity, or replication stress management sufficiently to alter its drug response.

Finally, not all resistance mechanisms require restoration of the original DDR vulnerability at all. Reduced *PARP1* trapping through *PARP1* alterations, restoration of PARylation following loss of *PARG*, and changes in replication stress signaling can decrease PARPi activity while HR deficiency persists [28,37]. More broadly, resistance may also arise through mechanisms that alter effective drug exposure or reduce target dependence, including decreased drug influx, increased efflux, enhanced drug inactivation or degradation, and overexpression or modification of the therapeutic target. These mechanisms may operate independently of, or cooperate with, changes in DDR pathways, further emphasizing that loss of therapeutic sensitivity does not necessarily imply restoration of the original DNA repair defect [28,37]. These mechanisms are particularly important for interpreting treatment failure: clinical resistance to a DDR-targeted agent does not automatically demonstrate functional restoration of the defective repair pathway. Resistance may instead arise by circumventing the pharmacological mechanism that exposes that vulnerability. Consequently, the evolutionary trajectories leading away from DDR-directed sensitivity can be divided conceptually into at least three classes: restoration of the original repair function, compensatory rewiring that permits survival despite incomplete restoration, and drug-specific escape that reduces the therapeutic exploitation of an otherwise persistent vulnerability.

Ionizing radiation should be considered within the same evolutionary framework. Radiotherapy generates a broad spectrum of DNA lesions, with DNA double-strand breaks representing a major cytotoxic lesion and HR and non-homologous end joining providing principal routes of repair. Repeated or fractionated irradiation can therefore impose strong selection on heterogeneous tumor populations, enriching pre-existing or treatment-adapted clones with greater capacity to sense, tolerate, or repair radiation-induced DNA damage [38,39]. Importantly, radiotherapy can also leave its own genomic record: longitudinal and pan-cancer analyses have identified radiation-associated increases in small and large deletions and characteristic mutational patterns consistent with error-prone double-strand break repair [40]. Thus, although the molecular routes to radioresistance are broader than DDR restoration alone and also involve hypoxia, cell-state plasticity, and microenvironmental adaptation, the central principle developed here also applies to radiotherapy: ionizing radiation can both reshape the genomic landscape and select for tumor cell populations with altered DDR competence. Consequently, a DDR state measured before irradiation may not fully represent the repair phenotype of a subsequently recurrent or re-irradiated tumor.

Taken together, these mechanisms portray therapeutic resistance as an evolutionary search for viable solutions rather than a predetermined molecular program. Strong selective pressure imposed by platinum compounds and PARPi repeatedly favors restoration or compensation of genome maintenance functions, but the molecular route taken depends on the biological constraints of each tumor and each subclone. The resulting state may therefore differ substantially from both the treatment-naïve tumor and the simple designation implied by its original DDR genotype. Crucially, however, evolutionary change can operate in the opposite direction as well. If tumors can restore or bypass a pre-existing repair defect, ongoing genomic instability, clonal diversification, and treatment pressure can also generate new DDR deficiencies that were absent at diagnosis. This reciprocal trajectory—the acquisition rather than the loss of genomic vulnerability—represents the second dimension required to understand DDR as a dynamic therapeutic state.

## 4. The Opposite Evolutionary Direction: Acquisition of New DDR Vulnerabilities During Tumor Progression

**Figure 3:**
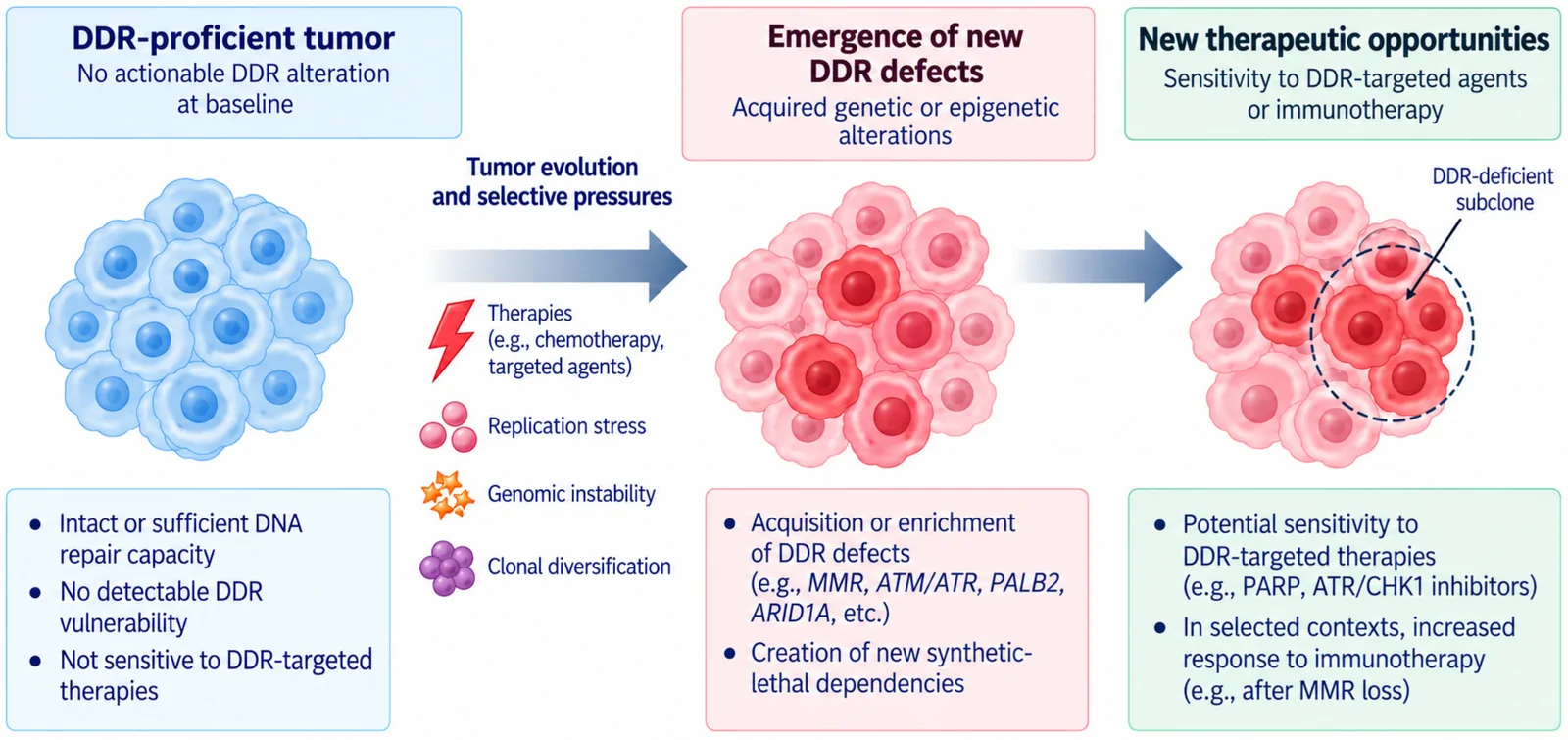
Emergence of new DDR vulnerabilities during tumor evolution. Tumors that are initially DDR-proficient may acquire or enrich genetic and epigenetic repair defects through treatment pressure, replication stress, genomic instability, and clonal evolution. These changes can generate new synthetic-lethal dependencies and potentially create therapeutic opportunities that were absent at baseline. Figure created with the assistance of ChatGPT 5.5 (OpenAI). The scientific content, final review, and validation were entirely performed by the Authors.

The evolutionary plasticity of DDR is intrinsically bidirectional. While restoration or bypass of a pre-existing repair defect can extinguish a therapeutic vulnerability, tumor evolution may also generate new repair deficiencies that were absent, undetectable, or biologically irrelevant at diagnosis. This possibility has received considerably less attention than acquired resistance to platinum compounds or PARP inhibitors (PARPi), partly because precision oncology has traditionally been organized around the identification of baseline actionable alterations. However, cancer progression is accompanied by continuous genetic diversification, replication stress, chromosomal instability, epigenetic remodeling, and repeated therapeutic bottlenecks, all of which can modify the architecture of genome maintenance. Consequently, DDR proficiency at diagnosis should not necessarily be interpreted as a permanent negative biomarker. A tumor that initially lacks an exploitable repair defect may evolve toward a state in which one repair pathway becomes impaired and survival becomes increasingly dependent on the remaining components of the DDR network. Such evolution can simultaneously generate a new molecular phenotype and a new synthetic-lethal opportunity. This evolutionary trajectory from baseline DDR proficiency to the emergence of new, potentially actionable repair vulnerabilities is schematically illustrated in Figure 3.

Perhaps one of the clearest demonstrations that treatment can actively generate a new repair phenotype comes from the interaction between alkylating agents and mismatch repair (MMR). Acquired MMR alterations after temozolomide exposure were initially characterized in glioblastoma, where recurrent tumors can acquire *MSH6* mutations and a hypermutator phenotype under treatment selection [38,39]. Importantly, this phenomenon has subsequently been deliberately investigated in colorectal cancer as a potential therapeutic strategy rather than simply as a mechanism of resistance. In the ARETHUSA study, patients with *MGMT*-silenced, initially MMR-proficient metastatic colorectal cancer received temozolomide with the explicit rationale of promoting a hypermutated state potentially capable of increasing susceptibility to immune checkpoint inhibition [40]. Longitudinal analysis of tissue and circulating tumor DNA demonstrated the emergence of a characteristic temozolomide-associated mutational signature, increased tumor mutational burden, and recurrent treatment-emergent alterations affecting MMR genes. Remarkably, the *MSH6* p.T1219I alteration was detected in tissue and/or ctDNA in 16 of 17 evaluable cases, demonstrating reproducible evolutionary selection of a repair-related genotype under pharmacological pressure [40,41,42,43]. Although the subsequent clinical benefit from pembrolizumab was limited and this strategy cannot yet be considered established therapeutic practice, ARETHUSA provides an important proof of principle: a DNA repair state can be pharmacologically reshaped during treatment, and longitudinal molecular assessment can identify a biologically relevant phenotype that was absent from the baseline tumor [43].

The implications extend beyond MMR. Loss of tumor suppressor genes involved directly or indirectly in genome maintenance can occur during clonal progression and alter the network of repair dependencies available to cancer cells. *ATM*, *ATR* pathway components, *PALB2*, *RAD51* paralogs, Fanconi anemia genes, and chromatin-remodeling proteins can influence the processing of double-strand breaks, stalled replication forks, interstrand crosslinks, and replication-associated lesions. Acquisition or enrichment of defects affecting these functions may increase dependence on complementary repair pathways, creating opportunities for synthetic-lethal targeting that were not present in the dominant baseline population.

More broadly, genomic instability can itself facilitate the emergence of additional vulnerabilities. Repair-deficient cells generate genetic diversity at an increased rate, and this diversity provides substrate for selection during therapy. Recent large-scale clinic-genomic analyses illustrate how inherited or pre-existing repair defects can influence the subsequent trajectory of somatic evolution. In *BRCA2*-associated breast cancer, for example, HRD has been linked to preferential acquisition of *RB1* loss under CDK4/6-inhibitor pressure, particularly when baseline hemizygosity has already reduced the evolutionary barrier to complete *RB1* inactivation [44]. Although *RB1* loss itself is not a newly acquired DDR deficiency, this observation is mechanistically informative because it demonstrates that the existing genome maintenance state changes the probability landscape of future genomic events. DDR alterations should therefore be viewed not only as biomarkers of current drug sensitivity but also as determinants of the evolutionary paths available to a tumor. A repair defect arising at one stage may promote additional genetic changes that subsequently create, modify, or extinguish other therapeutic dependencies.

This process can generate what might be considered nested vulnerabilities. Once one component of genome maintenance is lost, the surviving cancer population may become increasingly dependent on alternative repair mechanisms that were previously redundant. Such dependencies provide the biological foundation for synthetic lethality. *BRCA1/2*-deficient tumors represent the canonical PARP-dependent state, but analogous relationships are increasingly being explored involving *ATR*, *WEE1*, *POLQ*, *USP1*, *DNA-PK*, and other regulators of replication stress and repair [45,46,47]. Importantly, these dependencies themselves may change during tumor evolution. For example, alterations acquired during resistance to one DDR-directed therapy may create a different liability: *PARG* loss can contribute to PARPi resistance, yet experimental models indicate that *PARG*-deficient *BRCA2*-mutant cells become highly dependent on EXO1- and FEN1-mediated processes, generating a potentially exploitable secondary synthetic-lethal state [48]. Similarly, HR-deficient tumors may remain dependent on *USP1*-mediated stabilization of replication-associated repair processes even after developing resistance to PARP inhibition, and pharmacological *USP1* inhibition has demonstrated activity in PARPi-resistant preclinical models [49]. These observations suggest that therapeutic resistance does not necessarily represent the disappearance of vulnerability; in some circumstances, it represents its reconfiguration.

Epigenetic and transcriptional evolution may further generate or reveal DDR deficiencies without requiring new DNA sequence alterations. DNA methylation, changes in chromatin accessibility and histone modifications can alter the transcriptional state of repair genes, while regulatory non-coding RNAs, including microRNAs and long non-coding RNAs, may further modulate their expression and thereby influence pathway competence. Such mechanisms are particularly relevant because, unlike irreversible genomic deletions, these regulatory states may be reversible and heterogeneous across tumor populations. Consequently, the same tumor genotype may support different repair phenotypes at different disease stages. This plasticity complicates attempts to classify cancers permanently as DDR-proficient or DDR-deficient and reinforces the need to distinguish genetic potential from functional execution. It also suggests that acquired DDR vulnerabilities may occasionally be transient: a therapeutic window could emerge during one evolutionary phase and disappear after subsequent selection. From this perspective, timing becomes an integral component of biomarker actionability.

A further conceptual distinction is therefore necessary. Acquisition of a DDR alteration is not synonymous with acquisition of a therapeutically exploitable DDR vulnerability. A newly detected mutation in an HRR gene may be monoallelic, subclonal, functionally neutral, or insufficient to disrupt the pathway. Similarly, an increase in a genomic scar score during progression does not by itself establish sensitivity to a specific DDR-targeted therapy. The relevant evolutionary transition occurs only when the newly acquired molecular event produces a functional repair deficit of sufficient magnitude to generate a selective dependency. This requirement is particularly important when interpreting broad next-generation sequencing panels, which increasingly identify variants in large numbers of DDR-associated genes but cannot independently determine whether the corresponding pathway is functionally compromised. The distinction between DDR alteration, DDR deficiency, and DDR dependency should therefore remain explicit throughout clinical interpretation.

This problem becomes particularly relevant in gastrointestinal malignancies. Pancreatic, colorectal, gastric, and biliary tract cancers frequently undergo prolonged treatment with DNA-damaging agents—including platinum compounds, fluoropyrimidines, topoisomerase inhibitors and, in selected settings, alkylating agents—against a background of considerable spatial and temporal heterogeneity. The molecular profile obtained from the diagnostic specimen may consequently differ substantially from that of resistant metastatic populations months or years later. The ARETHUSA experience in colorectal cancer provides direct evidence that treatment-emergent repair alterations can be captured by ctDNA [42], while studies across gastrointestinal cancers increasingly identify heterogeneous HRR alterations and HRD phenotypes whose functional significance cannot always be inferred from archival tissue alone. In this setting, longitudinal molecular profiling should not be conceived solely as a strategy to identify mechanisms of resistance to the previous treatment. It may also reveal new vulnerabilities created by the evolutionary process itself.

The dynamic DDR model therefore extends beyond the simple transition from sensitivity to resistance. During its lifetime, a cancer may move repeatedly between different states of repair competence and dependency: an initially repair-proficient clone may acquire a DDR defect; treatment may selectively exploit that defect; resistant populations may subsequently restore the compromised pathway or bypass its requirement; and the resulting evolutionary solution may expose a different dependency elsewhere in the genome maintenance network. Rather than a binary trajectory from HRD to HR proficiency, DDR evolution may thus resemble a changing landscape of vulnerabilities constrained by genotype, epigenetic state, replication stress, treatment exposure, and clonal competition. This perspective has a major practical consequence: the absence of a DDR vulnerability at baseline does not prove its absence later, just as its presence at baseline does not guarantee its persistence. Determining when such vulnerabilities emerge, whether they are functionally active, and how long they remain therapeutically exploitable requires moving beyond static molecular classification toward longitudinal assessment of tumor evolution.

## 5. From Static Biomarkers to Longitudinal DDR States: Measuring an Evolving Therapeutic Vulnerability

If DDR competence can change during tumor evolution, its clinical assessment should ideally capture not only the molecular architecture inherited from previous evolutionary states but also the repair functions operating at the time a therapeutic decision is made. Current biomarker strategies only partially accomplish this objective because they interrogate different biological layers of DDR. Pathogenic variants in HRR genes identify potentially causal alterations; genomic instability scores and mutational signatures quantify the cumulative consequences of defective repair; epigenetic assays can reveal transcriptional silencing of repair genes; functional assays estimate the capacity of tumor cells to execute specific repair processes; and circulating tumor DNA (ctDNA) can provide repeated molecular snapshots of evolving tumor populations. These approaches should therefore not necessarily be regarded as competing methods for defining a single binary HRD status. Rather, they measure different temporal and functional dimensions of the same evolving biological system. Recent consensus recommendations for clinical HRD testing explicitly recognize the diversity of available biomarkers—including HRR-gene alterations, genomic scars, mutational signatures and epigenetic silencing—and emphasize important interpretative limitations when genomic instability measures are translated into clinical predictions [50]. The central challenge is consequently not simply to identify the most accurate HRD assay, but to determine which biological question each assay is capable of answering. The principal biomarker approaches, the biological dimensions they interrogate, and their temporal relevance within an evolving DDR state are summarized in Table 1.

Tumor sequencing remains an essential starting point because it can identify pathogenic germline or somatic alterations in *BRCA1*, *BRCA2*, *PALB2* and other DDR genes and establish the molecular context in which subsequent evolution occurs. However, the presence of a DDR gene alteration does not automatically establish functional pathway deficiency. Zygosity, loss of the wild-type allele, variant type and position, clonality, transcriptional consequences, and compensatory mechanisms can all influence whether a genomic alteration translates into an exploitable phenotype. Conversely, tumors without canonical HRR gene mutations may display genomic or functional evidence of HRD. A recent comprehensive clinical analysis integrating genetic, genomic and functional biomarkers across more than 500 tumor specimens demonstrated only low-to-moderate concordance among HRD assays; importantly, both *RAD51* testing and genomic instability scores identified HRD phenotypes in subsets of tumors lacking pathogenic HRR mutations [51]. These observations argue against a strictly gene-centric interpretation of DDR and support a layered model in which causal genotype, accumulated genomic phenotype, and current repair competence provide complementary rather than interchangeable information.

Genomic scar assays and mutational signatures occupy a particularly important but temporally complex position within this framework. Measures based on loss of heterozygosity, telomeric allelic imbalance, large-scale state transitions or genome-wide mutational patterns can recognize the cumulative consequences of previous HR failure and may therefore identify tumors in which the initiating defect is unknown or no longer readily detectable. Their persistence, however, makes them intrinsically less suited to establishing whether the corresponding deficiency remains functionally active after therapeutic selection. This limitation becomes especially relevant at progression. A tumor carrying a high HRD score after exposure to platinum or PARP inhibition may continue to display the chromosomal architecture generated during an earlier HR-deficient phase even if a resistant population has subsequently restored HR. Indeed, longitudinal profiling of PARPi-resistant *BRCA1/2*-altered breast cancers demonstrated that tumors harboring *BRCA* reversions retained HRD-associated mutational signatures despite showing functional HR competence according to *RAD51* assessment [52]. In this setting, repeating a scar-based assay may confirm the tumor’s evolutionary history without necessarily resolving the clinically more relevant question of whether the original vulnerability remains present.

Functional biomarkers potentially provide information closer to this current biological state. *RAD51* nuclear-foci formation following endogenous or induced DNA damage has emerged as one of the most extensively investigated functional readouts of HR competence. Because *RAD51* loading occurs downstream of several components of the HR pathway, its assessment can integrate the functional consequences of multiple upstream genetic and epigenetic events rather than relying on any single alteration. In ovarian cancer patient-derived xenografts, low basal *RAD51* foci strongly correlated with olaparib sensitivity and also appeared to be associated with platinum activity, supporting their potential role as a functional biomarker of homologous recombination deficiency [53]. This observation is particularly informative for the dynamic-state model: the same nominal *BRCA* genotype can coexist with different functional repair phenotypes. Functional testing is nevertheless not a universal solution. *RAD51* assays require sufficient proliferating tumor cells and adequate endogenous or experimentally induced DNA damage, are sensitive to tissue quality and sampling conditions, and principally interrogate HR rather than the broader DDR network. Moreover, spatial heterogeneity may allow HR-proficient and HR-deficient populations to coexist within the same patient. Functional assays should therefore complement, rather than simply replace, genomic measurements.

Longitudinal ctDNA analysis may provide the temporal axis that static tissue biomarkers inherently lack. Repeated tissue biopsies remain invaluable but are invasive, may be technically impossible, and typically sample only one lesion within a spatially heterogeneous metastatic disease. By contrast, ctDNA can be obtained serially and may capture DNA shed from multiple tumor deposits, allowing treatment-emergent alterations to be followed over time. Its value is particularly evident for *BRCA1/2* reversions. In longitudinal analysis of PARPi-resistant advanced breast cancer, reversion mutations were detectable in ctDNA before clinical progression in ten patients, demonstrating that molecular restoration of HR can become visible in the circulation before conventional evidence of treatment failure [52]. Pan-cancer sequencing has similarly detected *BRCA1/2* reversions in circulating cell-free DNA across multiple tumor types, including pancreatic and gallbladder cancers [54]. In pancreatic cancer, paired cell-free DNA analyses obtained during maintenance rucaparib demonstrated acquired *BRCA* or *PALB2* reversions in approximately 17% of evaluable patients progressing on therapy; importantly, reversions were associated with rapid PARPi resistance, markedly shorter survival and poor subsequent response to platinum rechallenge [55]. Thus, ctDNA can potentially transform resistance from a phenomenon recognized retrospectively after radiological progression into an evolutionary process that can be observed while it is developing.

However, ctDNA also illustrates why longitudinal genomics cannot by itself solve the functional problem. Detection of a reversion mutation strongly supports restoration of the affected gene, but absence of a detectable reversion does not establish persistence of HRD because resistance may arise through non-reversion mechanisms, epigenetic changes, replication fork stabilization or subclones shedding insufficient DNA into the circulation. Conversely, the variant allele fraction of an emerging reversion does not directly quantify the proportion of the total tumor burden that has become functionally HR-proficient. ctDNA should therefore be considered a dynamic genomic sensor, not a direct functional assay. Its greatest value may lie in revealing when the molecular composition of disease has changed sufficiently to justify renewed functional or tissue-based assessment. The integration of serial ctDNA with radiological evolution and treatment exposure could also help distinguish transient subclonal events from persistent evolutionary transitions.

Epigenomic assessment represents another potentially important component of this longitudinal framework. Promoter methylation of *BRCA1*, *RAD51C* and other repair-associated genes can contribute to HRD in the absence of coding mutations, while loss or reduction of methylation may restore gene expression and alter treatment sensitivity. Recent clinical recommendations therefore include epigenetic silencing among the molecular mechanisms relevant to HRD assessment [50]. Unlike permanent genomic scars, epigenetic states may change relatively rapidly and may differ between tumor populations, theoretically providing information intermediate between stable genomic history and immediate functional measurement. Nevertheless, technical standardization, thresholds defining biologically meaningful methylation, tumor purity and intratumoral heterogeneity remain substantial challenges. The same applies to emerging measurements of replication stress, replication gaps and fork protection, which may eventually complement *RAD51* by interrogating biological determinants of DDR-targeted sensitivity that cannot be captured by HR status alone. Contemporary models of PARPi resistance increasingly emphasize replication-gap biology and transcription–replication conflicts and have consequently proposed integrated biomarker frameworks combining genomic scars, *RAD51*, replication-associated measurements and ctDNA [56].

These considerations suggest that the future of DDR precision oncology may depend less on discovering another universal HRD classifier than on integrating biomarkers according to their temporal meaning. At baseline, germline and tumor sequencing can establish causal alterations, while genomic scars and mutational signatures can determine whether these alterations have produced a recognizable repair-deficient phenotype. During therapy, serial ctDNA can identify emerging resistant populations and new DDR alterations. At progression, contemporary tissue sampling—when clinically feasible—can reassess genotype, epigenetic state and tumor architecture, while functional measurements such as *RAD51* can determine whether HR deficiency persists. The recent integrated analysis combining tissue and liquid biopsy, genomic instability and functional *RAD51* measurements provides empirical support for precisely this complementary approach and shows that longitudinal ctDNA can identify *BRCA1/2* reversions after PARPi exposure that are associated with poor subsequent platinum response [51]. Rather than assigning a permanent label of “HRD-positive” or “HRD-negative,” the clinically relevant output could therefore become a time-stamped DDR state: the tumor was HR-deficient at baseline, remains functionally HR-deficient during treatment, has acquired evidence of HR restoration, or has developed a new repair dependency.

Such an approach may be especially relevant to gastrointestinal malignancies, where DDR-targeted strategies are clinically established only in selected molecular subsets and biomarker interpretation remains heterogeneous. Pancreatic cancer provides the clearest example. *BRCA1/2*- or *PALB2*-altered pancreatic cancers can display clinically meaningful platinum and PARPi sensitivity, yet acquired reversions can emerge after treatment and predict resistance to subsequent DNA-damaging therapy [55]. Pan-cancer analyses have also identified reversions in cholangiocarcinoma and gallbladder cancer, indicating that evolutionary restoration of HR is not confined to the classical *BRCA*-associated tumor types [54,57]. In colorectal cancer, as discussed above, serial ctDNA has demonstrated that treatment can generate new repair-associated alterations and mutational states, further supporting the feasibility of tracking DDR evolution in gastrointestinal disease. The challenge for these tumors is therefore not merely to expand DDR testing at diagnosis, but to establish when reassessment is biologically and clinically informative.

A longitudinal DDR framework should nevertheless avoid an important conceptual trap: more measurements do not automatically produce better treatment decisions. Serial sequencing, ctDNA, methylation assays and functional tests are clinically valuable only if a change in biomarker state can be linked to a therapeutic action. Prospective trials will therefore need to move beyond baseline biomarker stratification and incorporate predefined molecular reassessment points, ideally testing whether treatment adaptation based on emerging DDR states improves outcome. Trials could, for example, distinguish persistent HRD from HR-restored disease at PARPi progression, direct patients toward mechanistically appropriate subsequent therapies, or identify newly acquired synthetic-lethal dependencies before they become clinically dominant. Recent analyses of resistance to DDR inhibitors increasingly emphasize precisely this need to move from static genomic classifiers toward functional and longitudinal assessment of repair engagement, replication stress and tumor evolution [58].

Ultimately, the relevant unit of precision oncology may not be the biomarker, but the biomarker at a defined evolutionary time. A *BRCA2* mutation detected years earlier, a persistent HRD scar, a newly emerging reversion in ctDNA and absent *RAD51* foci do not provide redundant information: each describes a different layer of tumor biology and a different point along its evolutionary trajectory. Integrating these layers could allow clinicians to distinguish molecular ancestry from contemporary therapeutic dependency. The practical objective is therefore not continuous molecular surveillance for its own sake, but strategically timed reassessment capable of answering a clinically actionable question: what DNA repair vulnerability does this tumor possess now? Such a shift—from static molecular classification to longitudinal functional interpretation—may ultimately determine whether the expanding biological knowledge of DDR can be translated into more durable and evolutionarily informed precision oncology.

## 6. Translating Dynamic DDR States into Precision Oncology

The recognition of DDR as an evolving functional state has important consequences for precision oncology. A genomic alteration, the scar generated by that alteration, and the therapeutic vulnerability resulting from it do not necessarily persist for the same length of time. Accordingly, different classes of DDR biomarkers interrogate distinct biological dimensions. Pathogenic alterations in *BRCA1/2*, *PALB2*, *RAD51C*, *RAD51D*, *ATM*, or other repair-associated genes identify potentially relevant molecular events but do not invariably demonstrate pathway deficiency. Recent analyses of tumors arising in carriers of germline *RAD51C/D* pathogenic variants illustrate this distinction: fewer than 70% showed functional HRD, with retention of the wild-type allele accounting for a substantial proportion of HR-proficient tumors [59]. Conversely, genomic instability scores and mutational signatures capture the cumulative consequences of defective repair but may remain detectable after functional restoration. Functional biomarkers interrogate a more immediate biological layer. In the GeparOLA trial, *RAD51* testing further stratified tumors already selected as HRD by genetic or genomic criteria, with pathological complete response rates of 66.7% among *RAD51*-low tumors compared with 22.2% among *RAD51*-high tumors [60]. The relevant issue is therefore not which biomarker is intrinsically superior, but which biological question each biomarker is able to answer.

This framework introduces temporal validity as an additional dimension of biomarker interpretation. An HRD result obtained from a treatment-naïve primary tumor and the same result obtained after multiple lines of platinum therapy should not necessarily carry identical biological meaning. The former may reflect an active vulnerability, whereas the latter may partly represent molecular ancestry. *BRCA1/2* reversions can emerge during therapy, become detectable in circulating tumor DNA before clinical progression, and coexist with persistent HRD mutational signatures despite functional HR restoration [61]. Such observations support the concept of an evolutionary timestamp for molecular biomarkers: their interpretation should account not only for what was measured, but also for when it was measured relative to the selective pressures experienced by the tumor. This does not imply continuous comprehensive molecular profiling for every patient. Reassessment should instead be concentrated at evolutionarily informative clinical transitions, such as acquired resistance, prolonged exposure to a selective therapy, consideration of rechallenge with a mechanistically related agent, or evaluation of an alternative DDR-directed strategy. ctDNA is particularly attractive because it permits serial sampling and may reveal emerging resistant populations across multiple metastatic deposits. However, liquid biopsy remains primarily a genomic measurement rather than a direct assay of repair activity. Ideally, longitudinal ctDNA should therefore be integrated, when feasible, with contemporary tissue analysis and functional biomarkers capable of determining whether the pathway inferred from sequencing remains biologically deficient.

Future biomarker development should also move beyond universal definitions of HRD. Allelic context, clonality, tumor lineage, and functional state may substantially modify the biological consequences of apparently similar alterations. In the phase I TRESR study of the *ATR* inhibitor camonsertib, clinical benefit was greatest in tumors harboring biallelic loss-of-function DDR alterations, while longitudinal ctDNA molecular responses were strongly associated with clinical benefit [62]. Responses across different molecular contexts, including *ATM*, *BRCA1*, *BRCA2*, *RAD51C*, *CDK12*, and *SETD2* alterations, further illustrate that therapeutically relevant DDR dependencies extend beyond the classical *BRCA*–PARP relationship.

The emerging therapeutic landscape may therefore become increasingly network-based rather than gene-based. *ATR*, *WEE1*, *DNA-PK*, *POLQ*, *USP1*, and other regulators of replication stress and DNA repair are being investigated as potential targets in tumors with specific repair defects or in cancers that have escaped previous DDR-directed therapy [63,64,65,66,67,68,69,70]. Importantly, resistance to one DDR-directed agent should not automatically be interpreted as loss of all DDR-related therapeutic opportunities. A *BRCA*-reverted tumor, a tumor escaping through replication fork stabilization, and one becoming resistant through altered drug transport or *PARP1* modification may retain fundamentally different liabilities. The mechanism of resistance may itself become a biomarker of the next therapeutic vulnerability.

Gastrointestinal malignancies provide a particularly informative setting in which to test this framework. Pancreatic, colorectal, gastric, and biliary tract cancers display heterogeneous DDR alterations whose functional and predictive significance may vary substantially across tumor lineages and treatment histories. Rather than transferring HRD thresholds developed in ovarian or breast cancer directly to these diseases, prospective studies should establish whether similar genomic alterations generate equivalent functional deficiencies in different biological contexts. Transcriptional state, replication stress, co-occurring alterations, chromatin architecture, previous therapy, and the microenvironment may all modify the phenotypic consequences of a DDR alteration.

Several methodological challenges remain. Functional assays require analytical standardization and clinically validated thresholds; ctDNA sensitivity depends on tumor burden and shedding; methylation assays require robust definitions of biologically relevant silencing; and genomic signatures may vary according to sequencing platform and computational methodology [71,72]. Spatial heterogeneity further complicates interpretation because different metastatic lesions may simultaneously occupy distinct DDR states. The objective should therefore not be maximal molecular complexity, but the identification of the minimum combination of measurements capable of detecting a clinically actionable change in repair state.

Clinical trial design will need to accommodate this biological plasticity. Rather than assigning patients permanently to a molecular category defined at baseline, future studies could incorporate predefined longitudinal sampling, serial ctDNA, pharmacodynamic measurements, functional assays, and molecular reassessment at progression [73,74]. Treatment adaptation could then depend on whether the original deficiency persists, has been restored, or has been replaced by another dependency. Early clinical experience with camonsertib and functional *RAD51* assessment demonstrates the feasibility and potential value of integrating genomic and functional information into therapeutic development [59,60,62].

Ultimately, future biomarker frameworks should distinguish alterations associated with genomic instability from those that identify true therapeutic dependency. Not every mutation causes loss of function, not every loss-of-function event generates pathway deficiency, and not every pathway deficiency creates sensitivity to the same therapeutic agent. Integrating allelic status, clonality, genomic phenotype, functional repair competence, treatment history, and evolutionary timing may provide a more biologically faithful basis for DDR-directed precision oncology.

## 7. Conclusions and Future Perspectives

The success of DDR-directed therapies established one of the most compelling principles of precision oncology: loss of a DNA repair function can create a therapeutic dependency. Yet, cancer evolution adds an important temporal dimension to this paradigm. The alteration that initiated repair deficiency, the genomic scars produced during that period, and the resulting therapeutic vulnerability do not necessarily persist together. Genomic scars should therefore be interpreted for what they fundamentally are: records of evolutionary history. Their persistence can reveal that a tumor has experienced defective DNA repair, but it does not guarantee that the corresponding vulnerability remains active. Under endogenous and therapeutic selective pressures, tumors may restore a previously compromised pathway, bypass it through alternative mechanisms, or acquire entirely new repair deficiencies and dependencies. This distinction shifts the central question from whether a tumor is simply “HRD-positive” or carries a particular DDR alteration to whether the inferred repair defect is functionally present at the time treatment is being considered. It also suggests that resistance should not necessarily be viewed as the end of DDR-directed therapeutic opportunity. The evolutionary route through which resistance emerges may itself expose a new dependency. The challenge ahead is therefore not to abandon genomic biomarkers, but to place them within their biological and temporal context. Precision oncology has become increasingly proficient at identifying what alterations a cancer carries. The next step is to determine when those alterations became relevant, what functional state they produced, whether that state still exists, and what vulnerability may have replaced it.

A genomic scar can tell us where a tumor has been. The future of DDR precision oncology will depend on determining where its repair biology is now—and where evolution may drive it next.

## Figures and Tables

**Table 1:** Complementary biomarkers for longitudinal assessment of DNA damage repair states.

Biomarker Approach	Biological Layer Interrogated	What It Primarily Indicates	Temporal Meaning	Main Strengths	Main Limitations	Potential Role in Longitudinal DDR Assessment
Germline/tumor DDR-gene sequencing	Causal genotype	Pathogenic alterations in *BRCA1/2*, *PALB2* and other DDR genes	Relatively stable molecular ancestry, although clonal composition may evolve	Identifies potentially causal alterations; clinically established in selected tumor types; enables assessment of allelic status and emerging reversions	A mutation does not necessarily imply functional deficiency; interpretation depends on zygosity, clonality, variant type and biological context	Establish baseline molecular context and detect newly acquired or selected DDR alterations
Genomic scar assays (LOH, TAI, LST/genomic instability scores)	Accumulated genomic phenotype	Historical consequences of defective DNA repair	Cumulative/persistent	Captures previous HR failure even when the initiating defect is unknown; clinically validated in selected settings	May remain positive after functional HR restoration; limited ability to distinguish historical from current deficiency	Define whether a tumor has experienced HRD, but not necessarily whether HRD remains therapeutically active
Mutational signatures (e.g., HRD-associated signatures)	Historical mutagenic processes	Genome-wide footprints generated by defective repair	Retrospective/cumulative	Reconstructs repair processes beyond individual genes; may detect HRD without canonical *BRCA* alterations	Requires adequate sequencing and computational analysis; signatures may persist after functional restoration	Complement genomic scars in reconstructing the evolutionary history of repair deficiency
Epigenetic biomarkers (e.g., *BRCA1*/*RAD51C* promoter methylation)	Transcriptional regulation of repair	Potential silencing or reactivation of DDR genes	Potentially reversible/intermediate-term	Detects functional mechanisms not visible in DNA sequence; potentially responsive to tumor evolution	Assay standardization, thresholds, tumor purity and heterogeneity remain problematic	Identify acquired or lost repair defects not explained by sequence alterations
Functional HR assays (particularly *RAD51* foci)	Current repair competence	Ability of tumor cells to execute homologous recombination	Contemporary/near real-time	Measures pathway function downstream of multiple upstream alterations; can identify discordance between genotype/scars and actual HR competence	Requires adequate viable/proliferating tumor tissue and DNA damage; sampling and spatial heterogeneity; primarily evaluates HR rather than the entire DDR network	Determine whether HR deficiency is functionally present now, especially after treatment or at progression
Serial ctDNA profiling	Evolving genomic composition	Emergence or disappearance of resistant clones and DDR alterations, including reversions	Longitudinal/serial	Minimally invasive; repeatable; may reflect multiple metastatic sites; can detect molecular evolution before radiological progression	Indirect measure of function; sensitivity depends on tumor shedding and burden; absence of an alteration does not prove pathway competence or deficiency	Track emerging reversions and new DDR alterations and identify when tissue/functional reassessment may be warranted
Integrated longitudinal assessment	Genotype + genomic history + current function	Time-stamped DDR state	Evolutionary	Combines complementary information and explicitly separates molecular ancestry from present therapeutic dependency	Greater complexity; requires prospective validation and clinically actionable decision rules	Potential framework for treatment adaptation: persistent DDR deficiency, functional restoration, or acquisition of a new dependency

*BRCA*: Breast Cancer gene; ctDNA: circulating tumor DNA; DDR: DNA damage response; HR: homologous recombination; HRD: homologous recombination deficiency; LOH: loss of heterozygosity; LST: large-scale state transitions; *PALB2*: Partner and Localizer of *BRCA2*; *RAD51C*: *RAD51* paralog C; TAI: telomeric allelic imbalance.

## Data Availability

No new data were created or analyzed in this study. Data sharing is not applicable to this article.

## Abbreviations

The following abbreviations are used in this manuscript:

DNA	Deoxyribonucleic acid
DDR	DNA damage response and repair
HRD	Homologous recombination deficiency
BRCA1	BRCA1 DNA repair associated
BRCA2	BRCA2 DNA repair associated
PALB2	Partner and localizer of BRCA2
HRR	Homologous recombination repair
ADP	Adenosine diphosphate
PARP	Poly(ADP-ribose) polymerase
HR	Homologous recombination
RAD51	RAD51 recombinase
LOH	Loss of heterozygosity
TAI	Telomeric allelic imbalance
LST	Large-scale state transitions
CHEK2	Checkpoint kinase 2
DSB	Double-strand break
IHC	Immunohistochemistry
SBS3	Single-base substitution signature 3
Pt	Platinum
BRCA	Breast cancer susceptibility gene/protein family (BRCA1/2)
RIF1	Replication timing regulatory factor 1
PARPi	Poly(ADP-ribose) polymerase inhibitor(s)
ctDNA	Circulating tumor DNA
53BP1	p53-binding protein 1
SMARCAL1	SWI/SNF-related, matrix-associated, actin-dependent regulator of chromatin, subfamily A-like 1
FANCM	Fanconi anemia complementation group M
HLTF	Helicase-like transcription factor
MRE11	MRE11 homolog, double-strand break repair nuclease
CtIP	C-terminal binding protein-interacting protein
EXO1	Exonuclease 1
NHEJ	Non-homologous end-joining
TLS	Translesion synthesis
TP53BP1	Tumor protein p53-binding protein 1
PAXIP1	PAX-interacting protein 1
PTIP	PAX transactivation-domain interacting protein
CHD4	Chromodomain helicase DNA-binding protein 4
EZH2	Enhancer of zeste 2 polycomb repressive complex 2 subunit
PARP1	Poly(ADP-ribose) polymerase 1
PARG	Poly(ADP-ribose) glycohydrolase
MMR	Mismatch repair
MSH6	MutS homolog 6
MGMT	O6-methylguanine-DNA methyltransferase
ATM	Ataxia-telangiectasia mutated (ATM serine/threonine kinase)
ATR	Ataxia telangiectasia and Rad3-related (ATR serine/threonine kinase)
ARID1A	AT-rich interaction domain 1A
cGAS	Cyclic GMP-AMP synthase
STING	Stimulator of interferon genes
CHK1	Checkpoint kinase 1
RB1	Retinoblastoma 1
CDK4/6	Cyclin-dependent kinases 4 and 6
WEE1	WEE1 G2 checkpoint kinase
POLQ	DNA polymerase theta
USP1	Ubiquitin-specific peptidase 1
DNA-PK	DNA-dependent protein kinase
FEN1	Flap endonuclease 1
RAD51C	RAD51 paralog C
RAD51D	RAD51 paralog D
CDK12	Cyclin-dependent kinase 12
SETD2	SET domain containing 2, histone lysine methyltransferase
